# Ultrasound contrast agent Sonazoid for the diagnosis of hepatic epithelioid angiomyolipoma: a case report

**DOI:** 10.1186/s12876-021-02064-1

**Published:** 2021-12-20

**Authors:** Zhe Huang, Jun-yi Xin, Kai-yan Li

**Affiliations:** Department of Medical Ultrasound, Tongji Hospital, Tongji Medical College, Huazhong University of Science and Technology, 1095 Jiefang Avenue, Qiaokou District, Wuhan City, 430030 Hubei Province China

**Keywords:** Sonazoid, Hepatic epithelioid angiomyolipoma, Hepatocellular carcinoma, Case report

## Abstract

**Background:**

An angiomyolipoma usually occurs in the kidneys and rarely in the liver. Hepatic epithelioid angiomyolipoma (HEAML), a rare variant of angiomyolipoma, possesses malignant potential and mimics the imaging features of hepatocellular carcinoma. Sonazoid® (perfluorobutane microbubbles), a new contrast agent that facilitates hepatic parenchyma-specific Kupffer phase imaging on contrast-enhanced ultrasonography (CEUS), is useful for the detection and characterization of focal liver lesions.

**Case presentation:**

A 30-year-old man with HEAML underwent CEUS using Sonazoid®, in which new concepts for ultrasonography-based differentiation between HEAML and hepatocellular carcinoma were applied.

**Conclusions:**

This case report provides clinicians and radiologists with a reference for distinguishing HEAML from hepatocellular carcinoma based on CEUS using Sonazoid®.

## Background

Hepatic epithelioid angiomyolipoma (HEAML) is a rare variant of angiomyolipoma. Angiomyolipoma is a stromal tumor composed of smooth muscle cells, varying amounts of adipose tissue, and thick-walled blood vessels [1]. HEAML is mainly composed of epithelioid cells that possess malignant potential [2]. It can show striking cytologic atypia and multinucleation and may have local recurrence and metastasis [3]. Further, it can mimic the imaging features of hepatocellular carcinoma (HCC), such as hypo-enhancement in the portal phase in contrast-enhanced computed tomography (CT) [4], and therefore, it is difficult to clearly diagnose HEAML before surgery. The diagnosis and differential diagnosis of HEAML are very important owing to the differences in their respective prognoses. However, specific clinical manifestations and reliable laboratory test evidence for HEAML are lacking. Contrast-enhanced ultrasonography (CEUS) performed using a blood-pool contrast agent has demonstrated high sensitivity and specificity for characterizing focal liver lesions [5]. Sonazoid® (Daiichi-Sankyo, Tokyo, Japan; GE Healthcare, Milwaukee, WI, USA), a new contrast agent that permits hepatic parenchyma-specific Kupffer phase imaging on CEUS, leads to the observation of a persistent enhancement during the postvascular phase (Kupffer phase) for > 10 min after injection [6]. It is very useful for the detection and characterization of focal liver lesions and is now available in Japan, South Korea, Norway, and Taiwan [7].

This case study aimed to analyze the CEUS characteristics of HEAML using the Sonazoid contrast agent, and to devise new concepts to discrimination between HEAML and HCC.

## Case presentation

Physical examination of a 30-year-old man revealed hepatic space-occupying lesions, in the absence of chills, fever, nausea, vomiting, jaundice, and other symptoms. He was referred to our hospital for further treatment after a few days.

**Fig. 1:**
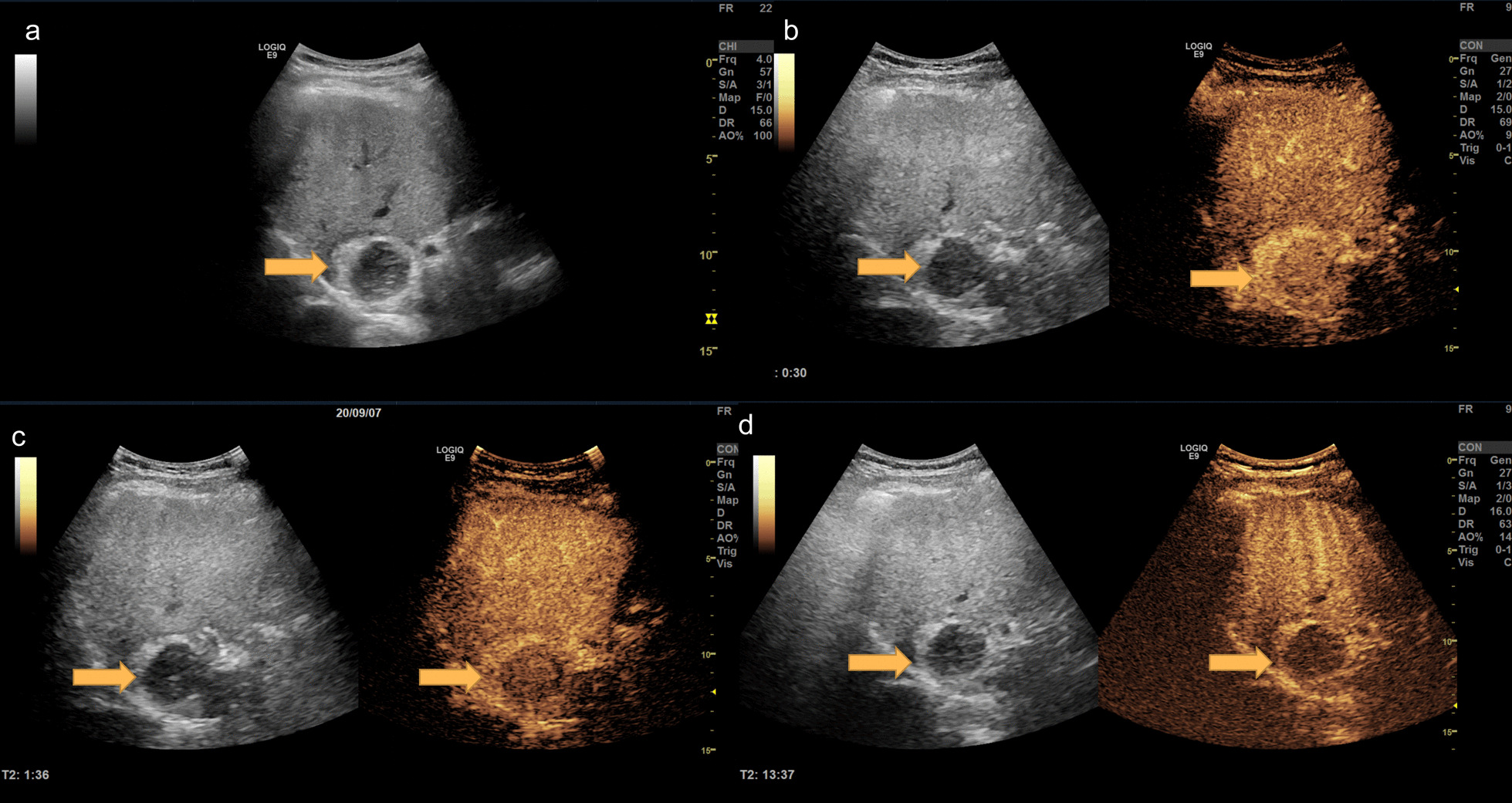
A liver mass discovered during physical examination in a 30-year-old male patient without chronic hepatitis B. The mass is estimated to measure approximately 4.6 cm × 3.6 cm and is located in the caudate lobe (**a**). On contrast-enhanced ultrasonography, the mass displays hyperenhancement after the injection of the contrast agent (**b**), contrast agent wash-out can be observed as a hypoenhancement at the center of the lesion, and the peripheral hyperechoic area remains hyperenhancing during the portal venous phase (**c**) and hypoenhancing during the Kupffer phase (**d**)

**Fig. 2:**
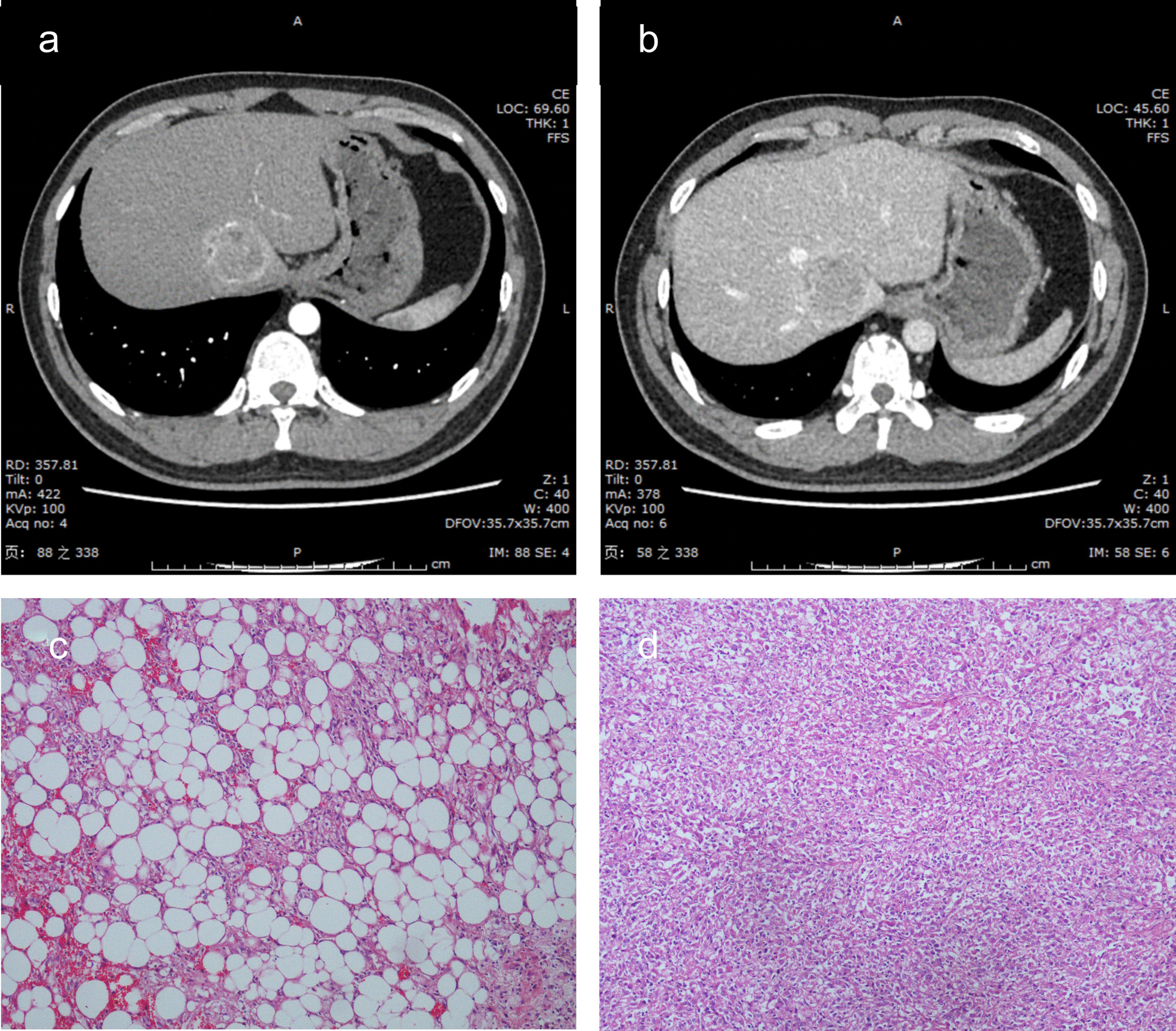
On enhanced computed tomography, the mass displays hyperenhancement after injection of the contrast agent (**a**) and early washout (**b**). Histopathological examination reveals that the lesion is a hepatic epithelioid angiomyolipoma (**c**, **d**)

Informed consent was obtained from the patient prior to the examination. The patient underwent CEUS at our inpatient department with a LOGIC E9 ultrasound scanner (GE Healthcare, Milwaukee, WI, USA). The level of low mechanical index (MI) in CEUS was 0.2. B-mode imaging revealed a hypoechoic zone (4.6 cm × 3.6 cm) in the caudate lobe of the liver, with a clear boundary, uneven internal echo, and hypoechoic zone surrounded by a hyperechoic ring. Color Doppler flow imaging did not find any obvious blood flow signal in the above-mentioned hypoechoic zone. CEUS revealed that the lesion was filled with the contrast agent, its center was moderately enhancing, and the peripheral area was hyperenhancing in the arterial phase. Contrast agent wash-out could be observed as a hypoenhancement at the center of the lesion and the peripheral hyperechoic area remained as a hyperenhancement in the portal venous phase. The central area exhibited further wash-out in the late vascular phase. The lesion showed a defect in the Kupffer phase (Fig. 1). Quantitative CEUS parameters reflecting tissue vascularity can be obtained from time-intensity curve (TIC) analysis. The fitted TIC of a region of interest can be obtained on a pixel-by-pixel basis using SonoLiver (TomTec, Germany, and Bracco, Italy). Quantitative analysis of CEUS showed that the contrast arrival time for the lesion was 8 s, the fall time for the lesion was 33 s, the mean transit time for the lesion was 15 s, and the time to peak for the lesion was 24 s. The peak intensity of the lesion was − 56 dB. The intensity of the lesion during the Kupffer phase was − 60 dB. The time to peak for the surrounding liver tissue was 13 s, the peak intensity of the surrounding liver tissue was − 62 dB, and the intensity of the surrounding liver tissue during the Kupffer phase was − 44 dB. The CEUS findings indicated that the liver lesions were benign but failed to clarify their specific nature. The patient underwent enhanced CT on the same day. The enhanced CT images showed a rounded, abnormal enhancement in the caudate lobe of the liver near the second hepatic hilum (4.4 cm × 4.1 cm), with obvious hyper-enhancement in the arterial phase, and wash-out in the portal and delayed phases (Fig. 2). The inferior vena cava and hepatic vein were compressed, and no obvious filling defect was observed, considering the possibility of HCC. During hospitalization, the patient underwent complete laboratory examinations within the next three days. He did not have a current or past history of hepatitis B virus and hepatitis C virus infection. The levels of alpha-fetoprotein (AFP), carcinoembryonic antigen, and sugar chain antigen were 1.72 ng/mL, 0.89 ng/mL, and 19–94.46 U/mL, respectively. The white blood cell count was 4.30 × 10^9^/L. The levels of alanine aminotransferase, aspartate aminotransferase, and total bilirubin were 19 U/L, 16 U/L, and 9.8 µmol/L, respectively.

We decided to perform laparoscopic hepatic caudate lobe resection for this patient after consultation with the hepatobiliary surgeon. Laparoscopic ultrasound exploration conducted during surgery revealed a soft, gray-brown mass measuring 3 cm × 3 cm located on the Spiegel’s lobe near the inferior vena cava. The Spiegel’s lobe was partially excised during surgery. The results of the histopathological examination showed that the lesion was a liver angiomyolipoma, and some areas were composed mainly of epithelioid and spindle-shaped cells (Fig. 2). The findings of immunohistochemical analysis were as follows: HMB45 ( +), smooth muscle actin ( +), cathepsin K ( −), S-100 ( −), SOX10 ( −), hepatocyte ( −), glypican-3 ( −), arginase ( −), AFP (-), GS ( +), cytokeratin (CK)19 ( −), EMA ( −), CK7 ( −), CK20 ( −), CD34 ( −), CD117 (c-kit 9.7) (-), CD117 (positive control) ( +), DOG1 ( −), DES ( −), caldesmon ( −), succinate dehydrogenase B ( +), CC21 ( −), CD23 ( −), CD35 ( −), and Ki-67 (LI approximately 3%). The results of fluorescence in situ hybridization were as follows: Epstein–Barr virus encoded RNA (EBER) chromogenic in-situ hybridization (CISH) ( −) and EBER CISH (positive control) ( −). The patient recovered well and was discharged after the surgery, and no tumor recurrence was observed at the 7-month follow-up.

## Discussion and conclusions

Hepatic angiomyolipoma is a rare hepatic stromal tumor, and HEAML is among its rare variants. Only a few more than 409 cases of EAML have been reported to date [8]. EAML is mainly composed of epithelioid cells and has a malignant tendency [9]. The differential diagnosis of HEAML and HCC is critical because of the differences in their treatment strategies and prognoses. The diagnosis of HEAML is often challenging because imaging alone cannot differentiate between HCC and HEAML in several instances.

HEAML mainly presents with hypoechoic nodules on gray-scale ultrasonography similar to HCC; this is consistent with our previous research [6]. The hyperechoic zone surrounding the HEAML lesion on gray-scale ultrasonography in this case may be a manifestation of the fibrosis of the liver parenchyma caused by long-term compression by the tumor. This has never been reported previously.

Hyperenhancement of the peripheral area in the arterial phase demonstrated by CEUS may be due to fibrosis of the liver parenchyma caused by long-term compression by the tumor. The deposition of extracellular matrix of liver fibrosis and the formation of hepatic sinusoidal capillaries lead to a state of high blood flow circulation. CEUS of HEAML showed rapid wash-out that is consistent with the performance of enhanced CT. Tumor cells have reduced interstitium, a rich vascular network, relatively thin tube walls, and increased blood flow, resulting in faster diffusion of the contrast agent, while the portal venous phase and late vascular phase are relatively lower than the liver parenchymal signal. The case study also used quantitative analysis software to assess the utility of Sonazoid in imaging HEAML and found that its performance was consistent with that of SonoVue in our previous study on HEAML [6]. The majority of the clinical evidence for the diagnosis of focal liver lesions with CEUS was obtained with SonoVue, while little is known about Sonazoid. It may be necessary to use this reagent for further studies on HEAML owing to the scarcity of published studies that have accurately correlated the enhancement mode and diagnostic accuracy of Sonazoid. Sonazoid has an additional Kupffer phase on hepatic CEUS. HEAML and HCC both exhibit hypo-enhancement in the Kupffer phase. In our case study, the enhancement intensity of HEAML in the Kupffer phase was − 62 dB. However, we studied 60 HCC lesions and found that the average enhancement intensity in the Kupffer phase was − 54 dB. The difference in the enhancement intensity of the HEAML tumor and surrounding liver tissue in the Kupffer phase was 17 dB, and that between HCC and the surrounding liver tissue was 6 dB. The difference between HEAML and HCC in the Kupffer phase and hyperenhancement of the HEAML peripheral area in the arterial phase may be their distinguishing factor. This requires more research of HEAML cases for verification.

Immunohistochemistry can help in differentiating HEAML from HCC. HEAML tumor cells express melanocyte markers (HMB45) and SMA to varying degrees, while epithelial cell markers (Pan-CK, EMA) are usually negative, which can help identify true epithelial tumors [10, 11].

This case report provides clinicians and radiologists with a reference basis for Sonazoid-based CEUS for HEAML. Future prospectively designed studies on the use of Sonazoid for HEAML cases should be done, which will help verify the characteristics of its differential diagnosis.

## Data Availability

This case report contains clinical data from the electronic medical record in the Nanjing Drum Tower Hospital. The datasets used during the current study are available from the corresponding author on reasonable request.

## Abbreviations

HEAML: Hepatic epithelioid angiomyolipoma
HCC: Hepatocellular carcinoma
CT: Computed tomography
CEUS: Contrast-enhanced ultrasonography
MI: Mechanical index
TIC: Time-intensity curve
AFP: Alpha-fetoprotein
EBER: Epstein–Barr virus encoded RNA
CISH: Chromogenic in-situ hybridization

## References

[1] Nonomura A, Mizukami Y, Kadoya M (1994). Angiomyolipoma of the liver: a collective review. J Gastroenterol.

[2] Tan Y, Xie XY, Li XJ, Liu DH, Zhou LY, Zhang XE, Lin Y, Wang W, Wu SS, Liu J (2020). Comparison of hepatic epithelioid angiomyolipoma and non-hepatitis B, non-hepatitis C hepatocellular carcinoma on contrast-enhanced ultrasound. Diagn Interv Imaging.

[3] Thway K, Fisher C (2015). PEComa: morphology and genetics of a complex tumor family. Ann Diagn Pathol.

[4] Jeon TY, Kim SH, Lim HK, Lee WJ (2010). Assessment of triple-phase CT findings for the differentiation of fat-deficient hepatic angiomyolipoma from hepatocellular carcinoma in non-cirrhotic liver. Eur J Radiol.

[5] Ye J, Xie X, Liu B, Zhang X, Wang W, Huang X, Lu M, Huang G (2017). Imaging features on contrast-enhanced ultrasound and clinical characteristics of hepatitis B virus-related combined hepatocellular-cholangiocarcinoma: comparison with hepatitis B virus-related hepatocellular carcinoma. Ultrasound Med Biol.

[6] Huang Z, Wu X, Li S, Li K (2020). Contrast-Enhanced Ultrasound Findings And Differential Diagnosis Of Hepatic Epithelioid Angiomyolipoma Compared With Hepatocellular Carcinoma. Ultrasound Med Biol.

[7] Moriyasu F, Itoh K (2009). Efficacy of perflubutane microbubble-enhanced ultrasound in the characterization and detection of focal liver lesions: phase 3 multicenter clinical trial. AJR Am J Roentgenol.

[8] Mao J, Teng F, Yuan H, Ni Z, Hong Fu, Liu C, Sun K, Zou Y, Dong J, Dong J (2018). 409 patients with hepatic epithelioid angiomyolipoma: a pooled analysis. Chin J Hepatobiliary Surg.

[9] Klompenhouwer AJ, Verver D, Janki S, Bramer WM, Doukas M, Dwarkasing RS, de Man RA (2017). IJzermans J: Management of hepatic angiomyolipoma: a systematic review. Liver Int.

[10] O'Malley ME, Chawla TP, Lavelle LP, Cleary S, Fischer S (2017). Primary perivascular epithelioid cell tumors of the liver: CT/MRI findings and clinical outcomes. Abdom Radiol (NY).

[11] Nguyen T, Terris B, Barat M (2020). Hepatic epithelioid angiomyolipoma mimicking hepatocellular carcinoma. Diagn Interv Imaging.

